# A Review of Natural Products Against Alcoholic Liver Fibrosis

**DOI:** 10.5152/tjg.2025.25154

**Published:** 2025-08-01

**Authors:** Shuangjiao Li, Tao Jiang, Wangdong Fan, Peiyao Liu, Lin Huang

**Affiliations:** Department of Pharmacy, West China School of Public Health and West China Fourth Hospital, Sichuan University, Sichuan, China

**Keywords:** Alcohol, chemical structure, liver fibrosis, natural product, pharmacological activity

## Abstract

Globally, alcoholic beverages top the charts in both sales and consumption rates. However, prolonged intake of these drinks often leads to hepatic injury, potentially progressing to more severe conditions such as hepatic fibrosis, cirrhosis, and in extreme cases, hepatocellular carcinoma. Over the past 2 decades, pharmacological research has increasingly validated the therapeutic potential of natural botanicals and their bioactive derivatives in the clinical management of hepatic fibrosis. In this review, anti-fibrotic secondary metabolites targeting alcohol-induced liver injury have been systematically analyzed, focusing on their botanical origins, pharmacodynamic profiles, structural characteristics, and mechanistic underpinnings. It provides some reference for developing natural products in the treatment of liver fibrosis in the future.

**Main Points**

- Alcoholic beverages are widely consumed globally, but long-term intake can lead to hepatic injury, including fibrosis, cirrhosis, and hepatocellular carcinoma.
- Natural botanicals and their derived medicinal formulations have shown efficacy in treating hepatic fibrosis over the past 20 years.
- This review focuses on active secondary metabolites in alcohol-induced liver fibrosis, covering their sources, pharmacological activities, and chemical structures.
- The findings provide a reference for developing natural products to treat liver fibrosis in the future.

## Introduction

Chronic hepatic disorders represent one of the most pressing global health challenges, contributing to nearly 1 million fatalities each year, primarily due to cirrhosis and its associated complications.^1^ Statistical evidence indicates that excessive alcohol consumption stands as the leading factor in cirrhosis development, responsible for 30% to 50% of global cirrhosis-related mortality.^2^As early as 2004, the World Health Organization highlighted that approximately 2 billion individuals globally consume alcohol, with around 75 million facing the threat of liver conditions linked to alcohol use, and this figure continues to grow annually.^1^

Alcohol contributes to this chronic injury in a multitude of ways. Firstly, alcohol promotes fat production in the liver and inhibits fat breakdown by modulating SREBP-1c, Egr-1, and Peroxisome proliferator-activated receptors (PPARs), causing lipid buildup and ultimately fatty liver (steatosis).^3^ Secondly, the metabolism of alcohol generates oxidative stress, triggers hepatocyte death, and impacts various non-parenchymal cells, leading to the release of multiple cytokines and sterile inflammation.^4^

There is growing evidence that these fibers are not permanent but reversible, and although current strategies for treating fibrosis still rely on eliminating the cause, there appears to be a huge market. According to data from Coherent Market Insights, worldwide healthcare expenditures for the clinical management of hepatic fibrosis amounted to $14.7 billion in 2022. Projections suggest an annual growth rate of 10.8% in these costs over the forecast period spanning from 2022 to 2030. However, these chemosynthetic drugs for a single target are a burden to patients because of their side effects, such as headaches, diarrhea, nausea, and rhinitis.^5^ The growing recognition of natural bioactive compounds in combating chronic liver diseases has positioned secondary metabolites from medicinal or dietary plants as a research hotspot in hepatology. In this comprehensive review, systematic searches were conducted across PubMed, Google Scholar, Web of Science, and China National Knowledge Infrastructure (CNKI) databases to collate anti-fibrotic phytochemicals targeting alcohol-induced liver fibrosis. Through multi-dimensional analysis of their pharmacological mechanisms, structural diversity, and plant origins, this work elucidates molecular pathways underlying their therapeutic effects while proposing innovative intervention frameworks for alcohol-associated liver fibrosis.

## Flavonoids

Flavonoids, as a class of compounds widely present in plants, foods, and fruits, have been developed into thousands of health supplements because of their beneficial and diverse pharmacological activities. In alcohol-induced liver fibrosis, flavonoids are also the most reported class of compounds; they treat fibrosis by resisting inflammation and oxidation, reducing extracellular matrix production and steatosis, and inhibiting stellate cell activation. Specific information is in Table 1.

Li et al^7^ revealed that isoorientin-2”-O-a-L-arabinopyranosyl mitigated alcohol-triggered fibrosis in mice by lowering transaminase levels, fibrosis biomarkers, and inflammation levels, enhancing alcohol metabolism, metalloenzymes, and antioxidant activity, promoting stellate cell apoptosis, and inhibiting TGF-β/α-SMA pathway,^6^ and the effect is close to that of the positive control (colchicine). Research findings demonstrate that combining compounds like genistein and taurine yields notable benefits in the clinical management of hepatic fibrosis. Protein analysis reveals distinct variations in the intensity of 4 specific protein peaks—WFDC 15A, LHRIF, NP-53, and COX8B—when comparing control groups with HSC-T6 cells subjected to combined treatments.^7^ However, the difference between co- and mono-use, model, and treatment groups should be added.

Naringenin and quercetin, widely distributed in plants and foods, both reduced liver fibrosis during ethanol-mediated tissue injury. In a separate study, the impact of 5 structurally distinct flavonoids—the effects of apigenin (a flavone), quercetin (a flavonol), naringenin (a flavanone), EGCG (a flavanol), and genistein—on alcohol-triggered liver damage in mice was assessed. Findings indicated that, at comparable doses, each of these compounds displayed protective effects against fibrosis, whereas no significant differences between them.^8^ Therefore, their structure-activity relationship should be further elucidated.

Fisetin is also particularly effective in improving histological changes; the low-dose group demonstrated superior efficacy compared to the standard treatment, silymarin. Notably, the fibrosis score showed a significant reduction from 9 in the model group to below 1.^9^ Further investigation into the underlying mechanisms is warranted for a comprehensive understanding.

Baicalin, a major component in Scutellaria baicalensis Georgi, has been shown through in vivo and in vitro studies to alleviate alcohol-related liver fat accumulation by enhancing fat breakdown and boosting lipid metabolism, ultimately reducing fibrosis. Mechanistically, Baicalin functions by inhibiting SREBP1c, resulting in the competitive interaction between PNPLA3 and ATGL.^10^

Diethylcarbamazine combined with hesperidin can block HSC activation, improve liver injury (AST, ALT, GGT, ALP, 4-hydroxyproline content), counteract hepatic oxidative stress, and augment hepatic antioxidants (MDA, GSH, NOx), and reduce release of hepatic IL6 and a-SMA.^11^

Beyond alcohol-induced models, mixed experimental methods were used to assess the anti-fibrotic potential of flavonoids. For example, a high-fat diet combined with alcohol was applied to investigate 7,8-dihydroxyflavone. These substances effectively reduced lipid accumulation, oxidative stress, inflammation, and fibrosis. Specifically, they worked through the AMPK/autophagy pathway, while 7,8-dihydroxyflavone utilized these mechanisms.^12^

On CCl_4_, the high-fat diet plus alcohol rat model, the tectorigenin treatment greatly decreased the histological average scores of hepatic fibrosis from 2.9 to 1.2 (max = 4); inhibited alanine aminotransferase (ALT), aspartate aminotransferase (AST), serum fibrosis markers; and improved antioxidant activity, and the ratio of albumin to globulin. Notably, Iris tectorum’s underground stems demonstrate substantial quantities of tectorigenin, with concentrations surpassing 1% according to phytochemical analyses.^13^

Puerarin, a key bioactive compound from *Pueraria lobata*, demonstrates protective effects against alcohol-induced liver injury. Studies show that it reduces hepatic lipid deposition and fibrosis progression, primarily by enhancing Bcl-2 mediated apoptosis in hepatic stellate cells (HSCs) to suppress fibrogenesis.^14^

Xanthohumol, the primary bioactive flavonoid in hops, is highly concentrated in beer. Interestingly, the type 1 diabetes rat treated with beer had more severe liver fibrosis and abnormal glucolipid metabolism than those treated with alcohol, but the addition of 10 mg/L xanthohumol to beer actually had a therapeutic effect.^15^

Research has demonstrated that specific flavonoid compounds exhibit anti-fibrotic effects in laboratory studies. Notably, compounds such as alpha mangostin and butein have shown the ability to suppress the activation of liver stellate cells triggered by alcohol and its metabolite acetaldehyde. These effects are mediated through multiple cellular pathways, including the TGF-β, as well as the NFκB, p38, and c-Jun N-terminal kinase (JNK) pathways, which are crucial in fibrotic processes.^16^

In conclusion, the above flavonoids have a similar nucleus and polyhydroxy substitution, but how they affect specific targets and structure-activity relationships still needs to be further studied.

## Terpenoids

Terpenoids refer to a class of natural compounds composed of olefins that are integer multiples of isoprene. Here, terpenoids with anti-fibrotic properties were classified according to the amount of their isoprene, as detailed in Table 1.

## Monoterpenes

We only found 2 monoterpenes against alcoholic liver fibrosis, gentiopicroside, and cannabidiol; their details are shown in Table 1.

Gentiopicroside halted the transition from chronic alcohol-induced liver fat accumulation to fibrosis, reduced lipid buildup in liver cells, and suppressed HSC activation. Mechanistically, it is related to the amp-activated protein kinase (AMPK) pathway.^17^

Cannabidiol activates ER stress by interacting with key molecules like PERK, ATF6, and IRE1, leading to the activation of the IRE1/JNK pathway and subsequent apoptosis. Importantly, this ER stress-induced cell death mechanism specifically targets activated HSCs. Evidence for this selective effect has been demonstrated in activated HSC lines derived from both humans and rats, as well as in mouse models in vivo.^18^

## Sesquiterpenes

Curcumol was reported to have anti-fibrotic activity, and their details are shown in Table 1. Curcumol, a bioactive component from Zingiberaceae plants, exhibits promise in addressing liver fibrosis. Research by Li et al^19^ revealed that curcumol treatment reversed severe liver fibrosis induced by CCl_4_ and alcohol in rat studies. This effect was linked to the suppression of the uPA/uPAR signaling pathway.

## Diterpenes

Acanthoic acid has demonstrated significant potential in mitigating alcoholic liver injury through a multi-faceted mechanism. It promotes the phosphorylation of LKB1-AMPK, a key regulatory step that activates SIRT1, contributing to a protective response against liver damage. This activation process is of utmost importance in blocking the IRAK1/4 signaling pathway, which holds a key position in managing lipid metabolism, hepatic fibrosis, and inflammation, thus helping to reduce the adverse impacts caused by prolonged alcohol exposure.^20^ Additionally, research has revealed that acanthoic acid can suppress lipin1 and lipin2 proteins, effectively blocking TLR4 activation triggered by ethanol in HSCs. This action not only blocks the initial trigger for inflammation but also disrupts the downstream IRAK-TRAF3-TAK1-NF-κB signaling axis, further reducing the inflammatory response characteristic of alcoholic liver disease.^21^

Cryptotanshinone demonstrated protective effects against ethanol-related liver damage by reducing fat accumulation, inflammation, and fibrotic changes. These effects were achieved by activating AMPK and Nrf2 pathways while inhibiting CYP2E1 activity.^22^

More information about acanthoic acid and cryptotanshinone is shown in Table 1.

## Triterpenes

Studies indicate that betulin effectively alleviates liver steatosis and halts fibrosis by blocking SREBP-1-regulated fatty acid production, as confirmed through both laboratory and animal studies.^23^

Lin et al isolated methyl helicterilate from Helicteres angustifolia, showing its potential to mitigate fibrosis by modulating mitochondrial pathways.^24^

Glycyrrhetinic acid, a key component of potentlini, has long been used in China to treat hepatitis and cirrhosis. According to Wang et al,^25^ this compound markedly suppresses HSC proliferation and collagen synthesis, while also minimizing the deposition of types I and III collagen in liver fibrosis caused by a mix of CCl_4_ and alcohol.

Significantly, research demonstrated that antrosterol ameliorated hepatic inflammation and fibrosis, evidenced by diminished fibrotic genes, concurrently with decreased serum AST enzymes. This compound influenced lipid metabolism positively by augmenting fatty acid catabolism while suppressing de novo lipogenesis, thereby enhancing the elimination of lipids and bile acids. Notably, its hepatoprotective profile included boosting antioxidant defenses through heightened alcohol dehydrogenase (ADH) and catalase (CAT) activity and downregulating CYP2E1, which contributed to reduced alcohol toxicity.^26^

The details of triterpenes against alcoholic liver fibrosis are shown in Table 1.

## Tetraterpenes

The terpenes with anti-alcoholic liver fibrosis are all carotenoids, as shown in Table 1. Astaxanthin-loaded liposomes, lutein, and meso-zeaxanthin C can decrease ALT levels and improve histopathological.^27^^-^29 However, these studies only confirmed their activity by phenotype but lacked in-depth mechanistic research.

## Phenylpropanoids

There are many phenylpropanoids with anti-alcoholic liver fibrosis, and their specific information is shown in Table 1.

Studies demonstrate that bioactive compounds in *Oenanthe javanica* alleviate alcohol-induced liver fibrosis by modulating fibrotic markers (reducing TGF-β/α-SMA, increasing MMP-1). Fermentation with *Lactiplantibacillus plantarum* significantly enhances its anti-fibrotic potential by boosting key phenolic constituents like p-coumaric acid.^30^

Chlorogenic acid, commonly found in coffee and tea, plays a role in regulating oxidative stress, inflammation, and fat accumulation. Research indicates that it also mitigates liver fibrosis and fat buildup in mice exposed to alcohol and a high-fat diet.^31^ Caffeic acid phenethyl ester in propolis also possesses antioxidant activity. It mainly regulates the downstream antioxidant genes by activating the Nrf2 transcription factor to achieve the therapeutic effect of fibrosis (score from 3.5 to 1.1), and the effect was better than that of the positive control vitamin E (1.3).^32^ In contrast, 4-O’-methylhonokiol alleviated hepatic fibrosis, necrotic lesions, and inflammation by abolishing CB1 receptor overexpression, whose pro-fibrotic effect has been repeatedly reported.^33^

Ferulic acid showed anti-fibrotic activity in alcohol and ∆polyunsaturated Fatty Acid (PUFA) treated rats by deregulating collagen, TIMPs, and promoting matrix degradation, while its analog methyl ferulic acid (the 4’ hydroxyl group on the benzene ring is replaced by a methoxy group) also attenuated liver fibrosis and HSC activation through the TGF-β1/Smad pathway.^34^

Xu et al^35^ employed computational approaches, including target prediction, simulation, and gene microarray analysis, to identify endothelin receptor B as a potential key target of schisantherin D in addressing the alcoholic liver disease. Further experimental validation revealed that lignans from *S*. *chinensis* effectively reduced fibrosis caused by ethanol and CCl_4_ in vivo. Additionally, schisantherin D mitigated cell damage in HL-7702 and LX-2 cells induced by EtOH and endothelin-1, while also lowering the levels of endothelin receptor B (ETBR), extracellular matrix components, and ET-1 secretion.^35^

## Coumarin, Polyphenol, and Stilbenoid

Fraxetin, ellagic acid, and polydatin have anti-fibrotic effects caused by alcohol. To date, fraxetin stands out as the only coumarin known to markedly reduce ethanol-induced liver fibrosis. Its protective effects on the liver are linked to improved ethanol metabolism and decreased oxidative stress. Remarkably, fraxetin at higher doses outperformed the positive control in efficacy.^36^

Polydatin, a natural stilbenoid, demonstrates protective effects against alcohol-induced acute liver injury in mice. It maintains normal hepatic architecture and significantly reduces fibrosis compared to control groups, potentially through modulation of metalloproteinases and enhancement of antioxidant defenses.^37^

## Quinones

Some quinones with anti-alcoholic liver fibrosis activity are shown in Table 1. Rhein and emodin, anthraquinone compounds derived from Chinese herbs such as Rheum palmatum, have demonstrated the ability to alleviate ethanol-triggered liver damage in mice. Studies indicate that these compounds significantly improved fibrosis-related histological changes, reduced α-SMA expression, and alleviated liver steatosis.^38^ ^39^

## Vitamins

Vitamin C (ascorbic acid) supplementation has been shown to reduce ethanol-triggered inflammation, endotoxemia, oxidative stress, and hepatic stellate cell activation in guinea pigs.^40^ Additionally, combining vitamin C with vitamin E resulted in a 20% greater reduction in liver fibrosis compared to using either vitamin alone.^41^

More information about vitamins is shown in Table 1.

### Alkaloids

Indole-3-carbinol (I3C), a compound abundant in cruciferous vegetables, alleviates alcohol-induced liver injury by modulating ethanol metabolism, reducing oxidative stress, and promoting collagen degradation to inhibit hepatic stellate cell activation.^43^ Betaine, a naturally occurring metabolite, demonstrates hepatoprotective effects in alcohol-related liver damage models, significantly improving fibrosis and steatosis.^42^ Betaine, a naturally occurring metabolite, demonstrates hepatoprotective effects in alcohol-related liver damage models, significantly improving fibrosis and steatosis.^43^ Both compounds show therapeutic potential, though their precise mechanisms require further investigation.

Caffeine, a key bioactive component in coffee and tea, has been indicated by rat studies to offer protection against fibrosis in the liver caused by alcohol consumption, as evidenced by biochemical and immunohistochemical analyses. Additional research proposes that caffeine exerts this effect through the suppression of the cAMP/CREB signaling cascade in HSCs.^44^

More information about the above compounds is shown in Table 1.

### Others

5-Hydroxymethylfurfural (5-HMF), recognized as a flavoring agent in numerous heat-processed items, is also present in plants, foods, and drinks.^45^ Research by Han et al^45^ demonstrated that 5-HMF markedly reduced fibrosis, inflammation, fat accumulation, and levels of hyaluronic acid (HA), collagen IV (CIV), laminin (LN), superoxide dismutase (SOD), and malondialdehyde (MDA) in mice exposed to CCl_4_ alcohol. Additionally, Cheng and Pan explored how ankaflavin and monascin can modulate PPAR-γ affected by ethanol, suggesting their potential as active compounds in monascus-fermented dioscorea for managing alcohol-related liver damage.^46^

Certain sulfur-containing organic compounds have demonstrated potential in reducing fibrosis. For instance, sulforaphane contained in broccoli and related vegetables exhibits anti-fibrotic effects. The compound exerts its effects by stimulating the Nrf2 antioxidant system, modulating the LPS/TLR4 pathway, and enhancing acetaldehyde breakdown.^47^ More details are shown in Table 1.

## Conclusion

Structurally, anti-fibrotic natural products were categorized into distinct classes including flavonoids, terpenoids, phenylpropanoids, and related derivatives, with particular emphasis on their structure-activity relationships in modulating fibrotic pathways. Among them, many compounds (fisetin, betulinic acid, polydatin, tetramethylpyrazine, and ferulic acid) have actually been confirmed to be active in other liver fibrosis models, but they still have many hurdles to overcome before becoming clinical drugs. In particular, the study of the mechanism of action still needs to be in-depth, and the direct targets should be elucidated. Second, the effective doses of some compounds (puerarin, propolis G, lutein, vitamin E, and vitamin C) in animal models are too high. When they are converted for application in humans, the doses are clearly not reasonable. Third, some experiments lacked positive controls, and it is hard to assess how effective they are.

In summary, natural compounds exhibit significant promise in treating alcoholic liver fibrosis. Their anti-fibrotic actions are primarily due to their anti-inflammatory, and antioxidant properties, their ability to curb steatosis, and diminish fibrogenesis. This overview offers valuable insights for future advancements and applications in anti-fibrosis drug development.

## Figures and Tables

**Table 1:** Natural Products Against Alcoholic Liver Fibrosis

Name	Formula	Model	Doses	Effects	References
Puerarin	C_15_H_10_O_6_	Rat, alcohol + pyrazole + Carbon tetrachloride (CCl_4_)	0.4 or 0.8 g/kg/day	↓ALT/AST and HSC apoptosis via ↓bcl-2 mRNA	14
Tectorigenin	C_16_H_12_O_6_	Rat, 30% alcohol + CCl_4_ + a high-fat diet	7.5, 15.0, or 30.0 mg/kg/day	↓ALT/AST/HA/LN, ↓LPO/MDA, and ↓hydroxyproline/collagen	13
Fisetin	C_15_H_10_O_6_	Mice, 50% alcohol	5 or 10 mg/kg/day	↑Liver function, ↑antioxidant defenses, ↑histological improvement, ↑ mitochondrial respiratory enzymes, and ↑MMP activity. Positive control: silymarin (25 mg/kg)	9
7,8-Dihydroxyflavon	C_15_H_10_O_4_	Rats, high-fat diet + 3%-15% alcohol	5 mg/kg/day	↓Fibrosis/oxidative stress (↓lipid peroxidation, ↑glutathione), ↓IL-1β (via ↓Nrf-2), and ↑iNOS mRNA	12
Taurine, epigallocatechin gallate, and genistein	C_2_H_7_NO_3_S C_22_H_18_O_11_C_15_H_10_O_5_	HSC-T6 cells	0.015-0.06, 0.0175-0.07, and 0.0035-0.014 mg/mL	↓Cell proliferation, ↓TGF-β1, and ↑MMP-2 mRNA Proteomics: WFDC15A/LHRIF/NP-53/COX8B	7
Apigenin, quercetin, naringenin, (−)−epigallocatechin gallate, and genistein	C_15_H_10_O_5_ C_15_H_10_O_7_ C_22_H_18_O_11_ C_15_H_10_O_5_	Mice, 50% alcohol	0.3 mmol/kg/day	↑Liver protection (↓alcohol damage), ↑serum markers, ↓hepatic lipid, ↑antioxidants, and ↓fibrosis/inflammation (genistein best ↓fibrosis)	8
Diethylcarbamazine and hesperidin	C_10_H_21_N_3_O C_28_H_34_O_15_	Rats, alcohol	50 mg/kg/day and 200/day	↓Fibrosis/↓4-hydroxyproline, ↓oxidative stress (↓MDA↑GSH/NOx), ↓IL-6/TGF-β1. Positive control: silymarin (100 mg/kg)	11
Isoorientin-2”-O-a-L-arabinopyranosyl	C_26_H_28_O_15_	Rat, alcohol	25, 50, or 100 mg/kg/day	↓ALT, ↓collagen ↓tissue damage, ↓lipid peroxidation, ↑antioxidants, ↓bcl-2 mRNA (→HSC apoptosis), and ↓α-SMA. Positive control: colchicine (1 mg/kg, i.g.)	6
Baicalin	C_21_H_18_O_11_	Rat, alcohol	200 mg/kg/day	↑TG, ↓hepatic vacuolization/steatosis, ↓fibrosis (↑SREBP1c→ ↑PNPLA3/FASN), and ↓PNPLA3-ATGL interaction→↓lipid hydrolysis	10
Xanthohumol	C_21_H_22_O_5_	Rats, 5% alcohol + streptozotocin + stout beer	10 mg	Fibrosis level↓ (3.85→1.78), ↓reticulin staining, ↑catabolic state, and glycogen↑ (4.68→22.09)	15
Alpha mangostin	C_24_H_26_O_6_	Acetaldehyde 100 μM for 24 h	10 and 20 μM	↓HSC growth/migration, ↓Ki-67/α-SMA/TIMP1, ↓ROS. Positive control: sorafenib (10μM)	16
Gentiopicroside	C_16_H_20_O_9_	Mice, 5% alcohol Lieber–DeCarli diet; LX2, TGF-β; Aml12, alcohol	40 mg/kg/day; 25-100 μM	↓Collagen I/α-SMA/TIMP1 (steatotic/fibrotic livers and LX-2 cells), ↑lipid metabolism, and ↓oxidative stress (AML12 cells)	17
Cannabidiol	C_21_H_30_O_2_	JS1 and HSCs	0-20 μM	↑Endoplasmic reticulum (ER) stress (↑PERK/IRE1/ASK1/c-Jun→HSC death) activate HSCs	18
Curcumol	C_15_H_24_O_2_	Rat, 6% alcohol + 40%-50% CCl_4_	12.5-50 mg/kg/day	↓LN/Col IV (rat model), ↓uPA/uPAR + MMP-13→↓fibrosis	19
Acanthoic acid	C_20_H_30_O_2_	Mice, diet containing 5% ethanol	20 or 40 mg/kg/day	↑Liver histology, ↓lipid/fibrosis/inflammation, ↓AST/TG (serum/hepatic), ↓ethanol/LPS, ↑p-LKB1/p-AMPK/SIRT1, and ↓IRAK1/4	20
Acanthoic acid	C_20_H_30_O_2_	HSC-T6 cells; mice, 5 g/kg ethanol	20 and 40 mg/kg/day; 10-20 μM	↓α-SMA and ↓ALT/TG/lipid (via ↓IRAK4/TLR4)	21
Cryptotanshinone	C_19_H_20_O_3_	Hepg2, AML12; mice, alcohol diet, for 4 weeks	Pre-treatment, 2.5 or 5 μM	↓Steatosis/inflammation/fibrosis (via ↑AMPK/Nrf2), ↑antioxidants/CYP2E1/TG/glutathione	22
Betulin	C_30_H_50_O_2_	Mice, 35% ethanol-containing diets and 5 g/kg ethanol; LX-2	20 and 50 mg/kg/day; 6.25-25 µM	↓LX-2 proliferation, ↓α-SMA, and ↓liver injury (via ↓TLR4/NF-κB axis)	23
Methyl helicterilate	C_17_H_24_O_4_	Rat, alcohol	16.72-66.90 mg/kg/day	↓Liver damage, ↓collagen, ↑ADH/ALDH, ↓cytokines, ↓TGF-β1/Smad2/3, ↑HSC apoptosis (mitochondrial)	24
Glycyrrhetinic acid	C_30_H_46_O_4_	Rat, 10% alcohol + 40% CCl_4_	6 mg, 3 times a week	↓HSC growth, ↓α-SMA, and ↓collagen	25
Antrosterol	C_28_H_44_O	Mice, 5% alcohol-containing diet	10 mg/day	↓Inflammation/AST/IL-1β, ↑lipid metabolism (↑β-oxidation and ↓lipogenesis ↑excretion), ↑antioxidants, ↓alcohol, ↑ADH/CAT, and ↓CYP2E1	26
Oleanolic acid	C_30_H_48_O_3_	Mice, alcohol + PM2.5; LX2	0.3 mM	↓IL-6/albumin, ↓steatosis/pericellular fibrosis, and ↑histological scores	27
Lutein	C_40_H_56_O_2_	Rat, ethanol	100 and 250 mg/kg/day	↓Liver damage (↓HP/LPO and ↑SOD/GSH)	28
Meso-zeaxanthin	C_40_H_56_O_2_	Rat, ethanol	50-250 mg/kg/day	↓Fibrosis and normalized AST/ALP/CD/CAT/GSH/hydroxyproline	29
Astaxanthin	C_40_H_52_O_4_	Mice, ethanol	Astaxanthin-loaded liposomes	↓Collagen/fibrosis/necrosis	30
Caffeic acid phenethyl ester	C_17_H_16_O_4_	Rat, alcohol + CCl_4_	3-12 mg/kg/day	↓TBil/AST/hydroxyproline, ↑GSH/SOD, ↓fibrosis (↓α-SMA ↑Nrf2). Positive control: vitamin E (10 mg/kg, i.p.)	33
Chlorogenic acid	C_16_H_18_O_9_	Rat, alcohol + high-fat diet	40 and 80 mg/kg/day	↓oxidative stress (↓CYP2E1 ↑Nrf2), ↓inflammation (↓TLR4/IL-6), ↓steatohepatitis/fibrosis,(↓srebp1/VEGF)	32
P-coumaric acid	C_9_H_8_O_3_	Rats, 30% ethanol	3.6 mg/kg/day	↓ALP, ↑albumin, ↓liver injury (↓TGF-β and ↑MMP-1)	31
Curcumin	C_21_H_20_O_6_	Rat, 40% ethanol; HSCs	200 and 300 mg/kg/day; 10-60 µmol	↓ALP/AST/ALT/collagen-I/α-SMA, ↓HSC proliferation, ↑HSC apoptosis (↑ER stress: PCNA/GRP-78/CHOP ↓TGF-β1/Smad)	35
Fraxetin	C_10_H_8_O_5_	Rats, ethanol	20 or 50 mg/kg/day	↓Fibrosis/ALT/AST, ↑liver structure, ↑ethanol metabolism (↑ADH/ALDH), ↓damage (↓MDA ↑GSH), ↓CYP2E1/IL-1β (↑HO-1)	37
Rhein	C_15_H_8_O_6_	Rats, 5% ethanol + CCl_4_	25 and 100 mg/kg/day	↑Liver structure, ↓ALT/MDA/PC-III/α-SMA	39
Emodin	C_15_H_10_O_5_	Mice, alcohol	50 mg/kg/day	↓ALT, ↓AST, ↓TG, ↓TC, ↓liver fat, ↓TBARS, ↓α-SMA, ↓collagen I, ↓CYP2E1, and ↑PPAR-γ	40
Alpha-tocopherol and ascorbic acid	C_29_H_50_O_2_ C_6_H_8_O_6_	Guinea pigs, ethanol	250 and 200 mg/kg/day	↓ALT, ↓GGT, ↓oxidative stress, ↓MDA, ↓CD, ↓protein carbonyls; collagen: ↓total collagen, ↓hydroxyproline; antioxidants: ↑SOD, ↑GR; expression: ↓TNF-α and ↓CYP2E1	42
Ascorbic acid	C_6_H_8_O_6_	Guinea pigs, ethanol	250 mg/kg/day	↓GGT, ↓MDA, ↓collagen I	41
Betaine	C_5_H_11_NO_2_	Rat, 5% alcohol + CCl_4_	2% betaine	↓TG, ↓oxidative stress, ↓fibrosis, ↓TGF-β, ↓MMP-2, and ↓TIMP-2	44
Caffeine	C_8_H_10_N_4_O_2_	Rat, ethanol	5, 10, or 20 mg/kg/day	↓ALT, ↓HA, ↓collagen, ↓α-SMA, ↓CIV, ↓cAMP pathway. Positive controls: colchicine (0.1 mg/kg)	45
Indole-3-carbinol	C_9_H_9_NO	hepatic stellate cell (HSC)	100, 200, and 400 μmol	↓MDA, ↓TGF-β1, ↓collagen, ↓HSC activation, ↓CYP2E1 (40.9%-51.8%), ↑ADH (1.6×), ↑MMP-1, and ↓TIMP-1	43

ADH, alcohol dehydrogenase. ALDH, aldehyde dehydrogenase. ALP, alkaline phosphatase. ALT, alanine aminotransferase. AMPK, amp-activated protein kinase. AST, aspartate aminotransferase. ATGL, adipose triglyceride lipase. α-SMA, α-smooth muscle actin. Bcl-2, b-cell lymphoma 2. CCl4, carbon tetrachloride. CAT, catalase. cAMP, cyclic adenosine monophosphate. CD, conjugated dienes. CHOP, c/ebp homologous protein. Col IV, collagen type iv. COX8B, cytochrome c oxidase subunit 8b. CTGF, connective tissue growth factor. CYP2E1, cytochrome p450 2e1. DPPH, 2,2-diphenyl-1-picrylhydrazyl. ECM, extracellular matrix. ER, endoplasmic reticulum. FASN, fatty acid synthase. GGT, gamma-glutamyl transferase. GR, glutathione reductase. GRP-78, glucose-regulated protein 78. GSH, glutathione. GSH-Px, glutathione peroxidase. HA, hyaluronic acid. HO-1, heme oxygenase-1. HSC, hepatic stellate cell. HSC-T6, rat hepatic stellate cell line. Hydroxyproline, hydroxyproline. IL-1β, interleukin-1β. IL-6, interleukin-6. iNOS, inducible nitric oxide synthase. IRE1, inositol-requiring enzyme 1. Ki-67, antigen ki-67. LHRIF, luteinizing Hormone Release-Inhibiting Factor. LN, laminin. LPO, lipid peroxidation. LPS, lipopolysaccharide. LX-2, human hepatic stellate cell line .MDA, malondialdehyde. MMP, matrix metalloproteinase. MPO, myeloperoxidase. NF-κB, nuclear factor kappa b. NOx, nitric oxide metabolites. NP-53, liver fibrosis-related protein. Nrf2, nuclear factor erythroid 2-related factor 2. p-LKB1, phospho-liver kinase b1. PC-III, procollagen type iii. PCNA, proliferating cell nuclear antigen. PERK, protein kinase r-like er kinase. PNPLA3, patatin-like phospholipase domain-containing protein 3. PPAR-γ, peroxisome proliferator-activated receptor gamma. ROS, reactive oxygen species. SIRT1, sirtuin 1.Smad2/3/4, smad proteins. SOD, superoxide dismutase.SREBP1c, sterol regulatory element-binding protein 1c. TBil, total bilirubin. TBARS, thiobarbituric acid reactive substances. TC, total cholesterol. TG, triglycerides. TGF-β1, transforming growth factor-β1. TIMP, tissue inhibitor of metalloproteinase. TLR4, toll-like receptor 4. uPA/uPAR, urokinase plasminogen activator/receptor. VEGF, vascular endothelial growth factor. WFDC15A, wap four-disulfide core domain 15a.

## Data Availability

The datasets used and/or analyzed during the current study were publicly available from the PubMed, Google Scholar, Web of Science, and CNKI databases.
